# A Machine Learning-Based Web Tool for the Severity Prediction of COVID-19

**DOI:** 10.3390/biotech13030022

**Published:** 2024-07-01

**Authors:** Avgi Christodoulou, Martha-Spyridoula Katsarou, Christina Emmanouil, Marios Gavrielatos, Dimitrios Georgiou, Annia Tsolakou, Maria Papasavva, Vasiliki Economou, Vasiliki Nanou, Ioannis Nikolopoulos, Maria Daganou, Aikaterini Argyraki, Evaggelos Stefanidis, Gerasimos Metaxas, Emmanouil Panagiotou, Ioannis Michalopoulos, Nikolaos Drakoulis

**Affiliations:** 1 Research Group of Clinical Pharmacology and Pharmacogenomics Faculty of Pharmacy, School oh Health Sciences, National and Kapodistrian University of Athens, 15771 Athens, Greece; dawn1980@hotmail.gr (A.C.); mkatsarou@pharm.uoa.gr (M.-S.K.); anniatsolakou@gmail.com (A.T.); vasilikieconomou99@gmail.com (V.E.); drakoulis@pharm.uoa.gr (N.D.); 2 Sotiria Thoracic Diseases Hospital of Athens, 11527 Athens, Greece; leonan7079@gmail.com (V.N.); ioa.nikolopoulos@gmail.com (I.N.); mdaganou@hotmail.com (M.D.); katrin.argyraki@gmail.com (A.A.); enl.stefanidis@gmail.com (E.S.); makmetaxas@yahoo.gr (G.M.); em_panagiotou@outlook.com (E.P.); 3 Centre of Systems Biology, Biomedical Research Foundation, Academy of Athens, 11527 Athens, Greece; emmanouil.christine@gmail.com (C.E.); margabrielatos@gmail.com (M.G.); dgeorgiou3@gmail.com (D.G.); 4 Department of Biology, National and Kapodistrian University of Athens, 15772 Athens, Greece; 5 Institute for Bioinnovation, Biomedical Sciences Research Center ‘Alexander Fleming’, 16672 Vari, Greece; 6 Department of Informatics and Telecommunications, National and Kapodistrian University of Athens, 16122 Athens, Greece; 7 Department of Neuroscience, Mayo Clinic, Jacksonville, FL 32224, USA; 8 School of Electrical and Computer Engineering, National and Technical University of Athens, 15773 Athens, Greece; 9 Department of Pharmacy, School of Health Sciences, Frederick University, 1036 Nicosia, Cyprus; hsc.mp@frederick.ac.cy

**Keywords:** SARS-CoV-2, severe COVID-19, age, sex, hypertension, obesity, cancer, machine learning

## Abstract

Predictive tools provide a unique opportunity to explain the observed differences in outcome between patients of the COVID-19 pandemic. The aim of this study was to associate individual demographic and clinical characteristics with disease severity in COVID-19 patients and to highlight the importance of machine learning (ML) in disease prognosis. The study enrolled 344 unvaccinated patients with confirmed SARS-CoV-2 infection. Data collected by integrating questionnaires and medical records were imported into various classification machine learning algorithms, and the algorithm and the hyperparameters with the greatest predictive ability were selected for use in a disease outcome prediction web tool. Of 111 independent features, age, sex, hypertension, obesity, and cancer comorbidity were found to be associated with severe COVID-19. Our prognostic tool can contribute to a successful therapeutic approach via personalized treatment. Although at the present time vaccination is not considered mandatory, this algorithm could encourage vulnerable groups to be vaccinated.

## 1. Introduction

The COVID-19 pandemic has stretched healthcare systems to their limits due to the increased cases requiring hospitalization, and to date, more than 7 million people have died from COVID-19 [1]. Although the majority of those infected are either asymptomatic or have mild symptoms, 5 to 20% rapidly deteriorate and require hospitalization [2]. Hence, it is essential to promptly recognize individuals at risk of their condition escalating to a severe stage, potentially resulting in death. In clinical medicine, early prediction of disease outcome (including mortality and ICU admission) would help in decision making and proper management of hospital resources, in order to relieve the already burdened health care systems [3]. Particularly, in countries where there is a large unvaccinated population, an increasing utilization of healthcare resources is observed due to the more severe manifestation of the disease [4].

The COVID-19 pandemic represents a unique opportunity to apply new prognostic tools with high sensitivity and specificity combining both novel and established biomarkers associated with infection and contributing to risk assessment [5,6]. In this direction, machine learning (ML) could prove to be a helpful tool, since as a field of artificial intelligence (AI), it aims at creating computer systems capable of learning and improving their performance based on prior knowledge and experience, using algorithms to improve their behavior and fulfil the task at hand [7,8]. ML can generate inductive reasoning, that is, generalizations derived from observing a group of cases, and can use large groups of data to discover new patterns based on their adaptive behavior [8]. The combination of simple data provided by the patient could lead to an early prediction regarding the severity of COVID-19. For instance, a meta-analysis of 120 systematic reviews found a significantly increased age-related risk of COVID-19 severity [9]. Older adults, especially those over the age of 65 years, are at a higher risk of developing severe symptoms and complications from COVID-19 [10], as their immune system weakens with age, making them more susceptible to infections [11]. Moreover, possible comorbidities obfuscate the effects of age on COVID-19 severity [9]. Comorbidities or underlying health conditions such as obesity, diabetes, hypertension, and heart disease, among others, may present important risk factors. These conditions weaken the immune system and make a person more vulnerable to infections and complications. People with underlying health conditions are at a higher risk of developing severe symptoms and complications from COVID-19 [12]. Other factors that may influence the severity of COVID-19 include sex, as men are more likely to develop severe symptoms and die from COVID-19 than women [10,13], genetics [14], and lifestyle factors, such as smoking [10] and alcohol consumption [15].

The aim of this study is to develop a webtool for predicting disease severity using information that can be easily and reliably provided by the patient. In this direction, machine learning (ML) could be a helpful means for prognosis. By employing ML and classical statistical analysis on data from unvaccinated COVID-19 patients, it is possible not only to identify key predictive factors but also to determine the degree that each of them contributes to the prediction. This leads to the creation of an accurate predictive model without the need for human intervention.

## 2. Materials and Methods

### 2.1. Data Collection

The present study included 344 COVID-19 patients who were admitted to the General Hospital of Thoracic Diseases “SOTIRIA” in Athens, Greece, from December 2020 to March 2022. SARS-CoV-2 infection was confirmed by qPCR, as this is the gold standard for viral detection [16]. Patients were initially divided into four categories according to the outcome of the disease, as follows: “No Hospitalization” (NH) (98 patients), “Simple Hospitalization” (SH) (106 patients), “Intubation and Survival” (IS) (56 patients), and “Intubation and Death” (ID) (63 patients). Patients were subsequently regrouped into two classes depending on the severity of the outcome. Patients with labels NH and SH were assigned to “Moderate COVID-19” (negative class containing 204 patients), while patients with labels IS and ID (admitted to ICU for intubation) were grouped into “Severe COVID-19” (positive class containing 119 patients). A questionnaire was used to collect data from NH patients, whereas the data for SH, IS, and ID patients were retrieved from their hospital medical records. The records of 2 patients were discarded due to missing medical history and those of 19 patients due to vaccination against COVID-19 prior to infection. A total of 122 independent medical characteristics were considered for statistical and ML analyses. The parameters studied included demographics, genetics, comorbidities, symptoms, and medical test results.

### 2.2. Statistical Analysis

Data analysis was conducted on all the records (age, health history, and medical records). Initially, 2 × 2 contingency tables were constructed with the observed frequencies. The two columns of the tables represent the “Moderate” and “ICU” patients, and the two rows are the two values of each categorical variable. Odds ratios (ORs) and 99% Confidence Intervals (C.I.s) were then calculated [17]. To determine the statistical significance of the effect of each variable, *p*-values were calculated using Pearson’s χ^2^ test [18] (0.01 significance level). The statistical analysis also involved subgroup and feature comparisons that were not used for machine learning. All statistical analyses were performed using IBM SPSS Statistics 29.0 [19].

### 2.3. Machine Learning

Our first goal on building the interpretable machine learning model is to remove from our dataset features that do not hold any classification information to help the model overcome the curse of dimensionality and create models that are easier to interpret. Towards that goal, the Variance Threshold algorithm from Scikit-learn v0.2.8 [20] was applied to remove features that were in a constant or a near-constant state (threshold = 0.01), as they would not provide any valuable information to our model.

**Figure 1:**
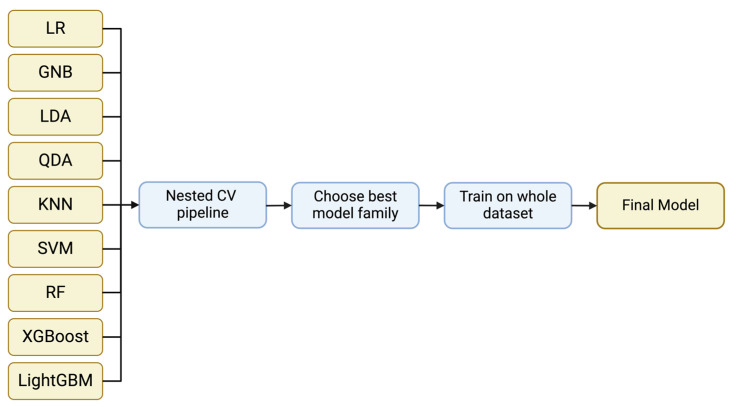
A general representation of the implemented pipeline. Nine different model families were evaluated using nested CV. Through the evaluation, the optimum model family is selected and the model is trained on the whole dataset to acquire the final model.

The classification model families used include Logistic Regression, Gaussian Naïve Bayes, Linear Discriminant Analysis, Quadratic Discriminant Analysis, k-Nearest Neighbor, Support Vector Machine and Random Forest from Scikit-learn v0.2.8 [20], XGBoost v2.0.0 [21], and LightGBM v4.1.0 [22] (Figure 1). These models cover a wide range of model families and approaches from linear models to quadratic and more complicated ones such as SVMs and decision tree-based models. This way, we can assess the performance of simple classifiers such as Logistic Regression to more state-of-the-art approaches such as XGBoost and LightGBM which can separate the feature space in more sophisticated and complex ways. If a simple model has a similar performance to a more advanced model, we ought to select the simple model to aim for a better generalization ability.

The small size of our dataset does not allow us to create distinct training, validation, and test sets to train and then assess the performance of our model on unseen data. Different approaches have been developed to tackle this issue and evaluate the performance of a model. The most widely used technique is the simple cross-validation (CV) experiment in which the dataset is split in k non-overlapping folds. k-1 of these folds are used to train the data and the final fold is used as a validation set, which evaluates the performance of the model by providing an unbiased estimate of the expected predicted error since the validation set’s data have not been used during the training process. A CV trial produces k different estimates of the selected model. This way, we can calculate an approximation of the performance of the model on unseen data when trained on the whole dataset.

It is important to note that the CV method is used as a technique to evaluate a specific instance of a model, tuned with certain hyperparameters [23], meaning parameters that control the learning process of the model and need to be predetermined before the training process. Therefore, an important part of the performance of a model is the selection of the appropriate hyperparameters for the specific problem. In our case, we need to be able to estimate the optimum hyperparameters and at the same time estimate the performance of the resulting model. When the same cross-validation procedure and dataset are used to both tune and select a model, it is likely to lead to an optimistically biased evaluation of the model performance. When we use the same dataset to tune the hyperparameters of the model but also estimate its performance, then the performance estimate will be biased, leading to highly optimistic results.

**Figure 2:**
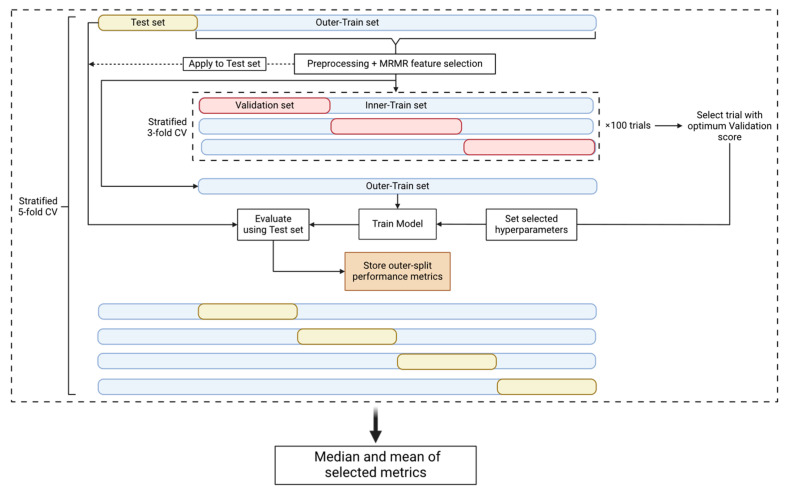
Nested CV pipeline. The nested CV is comprised of an outer-stratified 5-fold CV (test set and outer-train set splits) and an inner-stratified 3-fold CV (validation set and inner-train set splits). The dataset first is split into a test and an outer-train set. The train set is preprocessed and then used for MRMR feature selection. The parameters and the selected features are used to transform the test set. The outer-train set is then passed on to the inner CV where it is split into validation and inner-train sets. The inner CV is used for hyperparameter tuning and 100 trials/CVs are performed. We select the trial with the best validation score to use its selected hyperparameters. The model is then tuned with the selected hyperparameters and trained on the outer-train set. Finally, we evaluate the trained model using the test set and store the performance metrics. The procedure is performed for every fold of the outer CV and therefore we acquire 5 different metric values per nested CV round. The nested CV procedure is repeated 10 times to obtain 50 performance metric instances. The median and mean values of the metrics are calculated for the specific experiment.

In an attempt to prevent the introduction of any bias that could be derived from the use of the same datasets to perform both the hyperparameter tuning and the performance evaluation, nested cross-validation (nested CV) [24] (Figure 2) comprising an outer and an inner CV loop was used. The outer loop is used to estimate the performance of the model and the inner loop is used to estimate the optimal set of hyperparameters. This approach, even though it is very computationally expensive, minimizes the risk of the model overfitting the dataset and provides an estimate of the true tuned model’s performance on unseen data. Consequently, since the test set, which represents 20% of our data, is unknown to our model as it is used only for the final test of the tuned and trained model, the use of an external and independent test set is not necessary to evaluate the model’s performance.

In the outer CV loop, the dataset is split into a training and a test set. The former is used for preprocessing and feature selection, and later for hyperparameter tuning, while the latter is only used for the performance evaluation of the tuned model. Our pipeline used a stratified 5-fold outer CV. Once the data were split into the training and test set, the missing values of the training set were filled using the mean imputation technique [25]. The mean values that were used to fill the missing values of the training set were also used to fill the missing values of the test set. When the experiment parameters denoted that the dataset should be standardized, we standardized our features using the Standard Scaler algorithm [26] provided by Scikit-learn. The training and test sets were standardized separately. We then performed feature selection using the Maximum Relevance–Minimum Redundancy (mRMR) algorithm [27] (https://github.com/smazzanti/mrmr, accessed on 20 June 2024) to select the minimum number of optimal features using the training set. A range of N numbers of features (5, 10, 20, 30, and 35) were tested to evaluate the classification accuracy of a smaller group of features, and the training and test sets were reduced to the selected features. To increase the performance of the model for the minority class (positive class), the SMOTE algorithm [28] was applied only on the training set to oversample this class [29]. The last step of the training set preprocessing was the transformation of the features using the Min-Max Scaler algorithm [26] from Scikit-learn, which is performed only if the classification model was Logistic Regression, K-Nearest Neighbor, or Support Vector Machine [30]. Once the preprocessing and the feature selection steps were completed, the training set was fed into the inner CV loop.

The inner CV loop is used for hyperparameter tuning, as already mentioned. We used the Optuna v3.3.0 package [31] to perform 100 hyperparameter tuning trials using Matthew’s Correlation Coefficient (MCC) [32] as the tuning metric. Each trial is a stratified 3-fold CV that outputs a mean validation set MCC score that must be optimized [33]. Therefore, upon the end of the hyperparameter tuning procedure, Optuna provided the selected hyperparameters that produced the optimum validation score.

In the outer loop, the model was tuned using the selected hyperparameters and fitted using the training set. The test set was used to make predictions and the performance metrics reported were Balanced Accuracy, MCC, F1 score, Precision, Recall, and Specificity [34]. For one nested CV circle, 5 different values for each metric were calculated. The nested CV procedure described was performed 10 times to acquire a larger set of metric values. The median and mean values of each metric were reported, based on the 50 different outer-train and -test splits, for each experiment and model family.

### 2.4. Model Interpretability

SHAP analysis [35] was performed for the interpretation of the predictions of the selected model. The experiment’s parameters such as the number of features selected by mRMR, the model family, and the preprocessing steps already discussed, determine the flow of the SHAP analysis. Since we already knew the number of the desired features, we performed mRMR using the full dataset to reduce our dataset to the selected features. The SHAP analysis pipeline we employed performed a similar nested CV workflow. At the end of each outer-CV loop, a bar plot and a bee swarm plot were created to interpret the model, and that is why 5 bar plots and 5 bee swarm plots were obtained. The bar plots are used to outline the global feature importance while the bee swarm plots demonstrate how the feature values affect the model’s performance.

### 2.5. Website

Based on the optimized prediction model, a website was created in order to predict the outcome of the disease in new patients. Users fill in a form with the values of the following features: “age”, “sex”, “arterial hypertension”, “obesity”, and “cancer”. The website is deployed on an HTTPS protocol-verified Apache2 2.4.52 web server, which is hosted on a 16-core, 64 GB memory, Ubuntu 22.04 Linux system, and is available at https://www.michalopoulos.net/covid/ (accessed on 10 June 2024).

## 3. Results

### 3.1. Statistical Analysis

**Figure 3:**
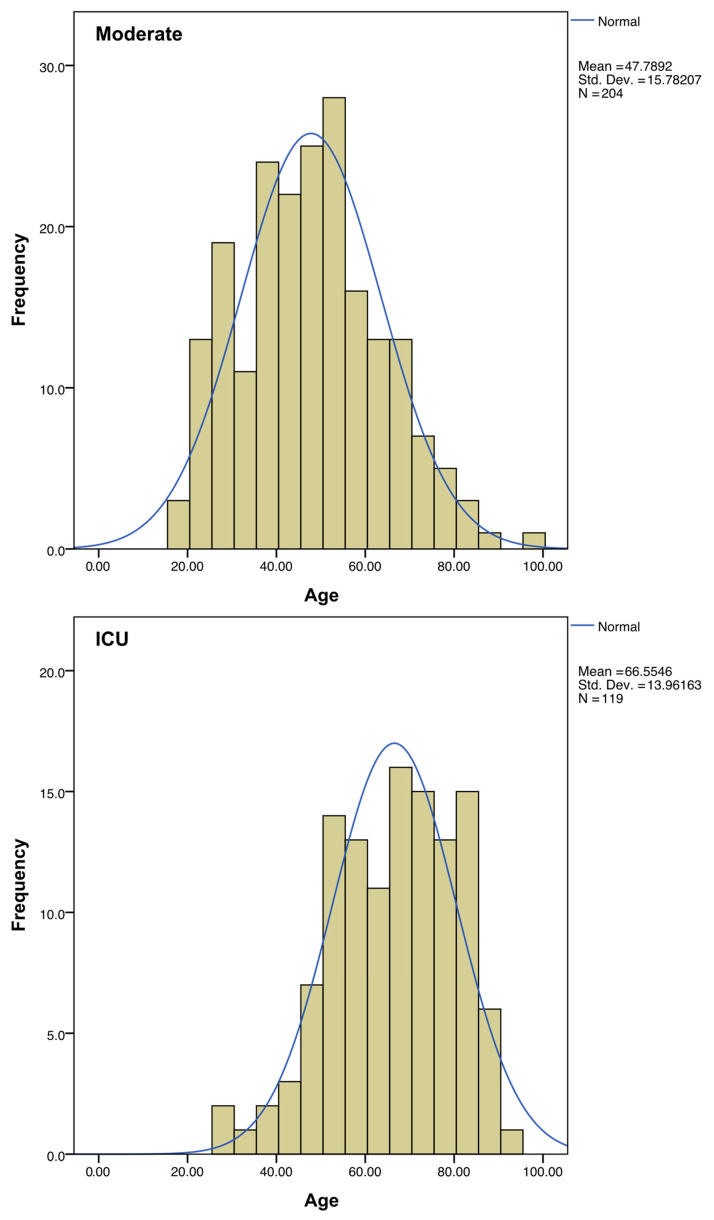
Distribution of ages among the “Moderate” and “ICU” subgroups. A normal distribution curve (in blue) was calculated to fit each histogram.

Regarding sex, the percentage of ICU-hospitalized patients is higher in men than in women. The mean age of patients admitted to ICU was almost 20 years higher than the average of the patients with moderate symptoms (Figure 3, Table 1).

The comorbidity with the largest statistically significant difference between moderate and intensive care patients was arterial hypertension (*p*-value < 0.001, OR 6.46). Obesity (*p*-value 0.001, OR 3.25), diabetes mellitus (*p*-value 0.001, OR 4.06), chronic obstructive pulmonary disease (COPD) (*p*-value 0.001, OR 11.94), and malignancy (*p*-value 0.001, OR 4.08) were also highly predictive of ICU admission. Dyslipidemia and atrial fibrillation also seem to have a slight aggravating role in our sample, although they have a higher *p*-value (0.009 and 0.003, respectively) than the previous ones. Thyroid disease, bronchial asthma, neurological disease, and coronary disease did not show a statistically significant effect on disease outcome (Table 2).

The mean ferritin level on the day of admission was three times higher in those who went on to develop severe disease than in those who did not require intensive care, and there was a statistically significant likelihood of ICU patients having a level above 800 ng/mL (norm: male: 12 to 300, female: 12 to 150). Hospital admission day values for D-dimers and glucose showed no statistically significant difference at the 99% CI level (Table 3).

### 3.2. Machine Learning

We took into account all classification metrics (Table S1), with a focus on Recall and Specificity. The chosen model should have high specificity to minimize false positive calls, while at the same time, we aim for a high recall so our model will be able to correctly identify most of the cases with a high risk of hospitalization. We concluded that the optimal model is Linear Discriminant Analysis (LDA) with mRMR as a feature selection method, five features retained in the dataset, and the features being scaled. SMOTE balancing was not applied.

**Figure 4:**
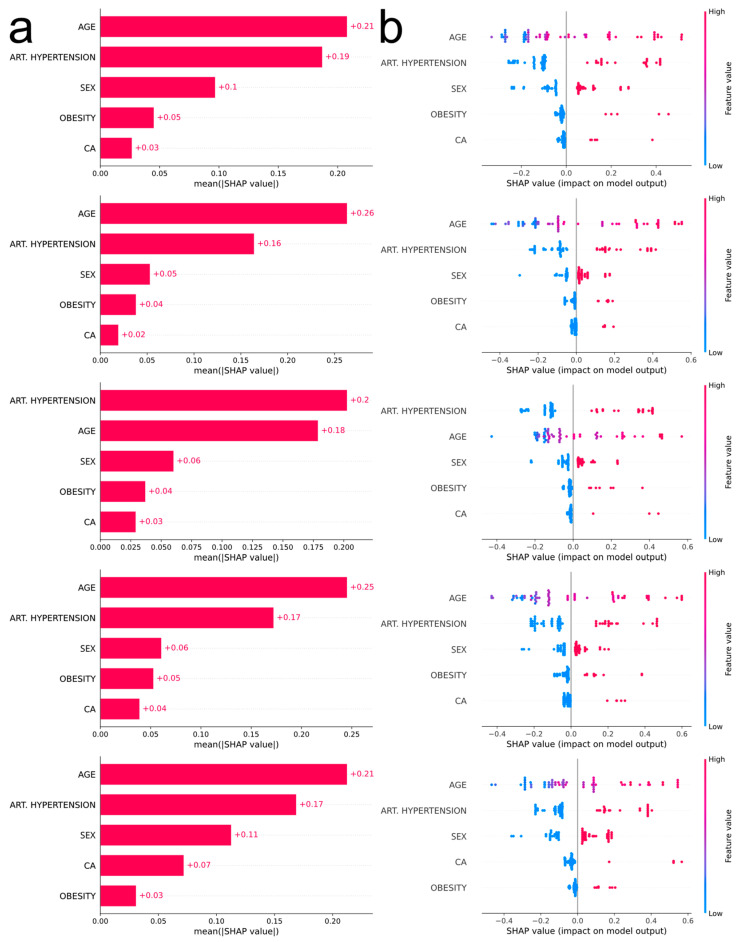
(**a**) Bar plots corresponding to each outer CV loop depicting the impact of the most influential features, in descending order, using absolute SHAP values. (**b**) Bee swarm plots corresponding to each outer CV loop depicting the impact of the most influential features, in descending order, using non-absolute SHAP values. Each point on the plot corresponds to one observation. The color scale represents the value of each variable for each observation. Negative values on the horizontal axis indicate a positive association of the feature with moderate COVID-19 prediction, while positive values indicate a positive association of the feature with severe COVID-19 prediction. For comorbidities, blue dots correspond to absence of the disease (0), whereas red dots correspond to the occurrence of disease (1). For sex, blue dots correspond to female (0) and red dots to male (1). Finally, age is depicted with a blue–red gradient (18–100 years old).

The most influential feature in four out of five outer CV loops is age (Figure 4a), with increased values being associated with severe COVID-19 manifestation (Figure 4b). The second most influential feature is arterial hypertension, the existence of which pushes the prediction towards a more severe outcome. According to the consensus of the five plots, the next most influential features are sex, obesity, and malignancy (males more prone to severe disease than females, obesity and malignancy associated with severe disease).

**Figure 5:**
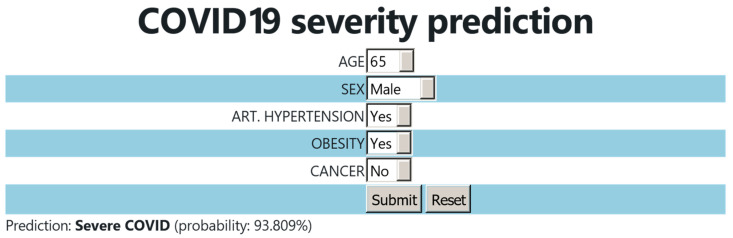
User interface of the COVID-19 severity prediction webtool.

Based on the constructed model, a user-friendly webtool for the COVID-19 severity prediction was developed. The users select the age and sex of the patient. They also provide an answer to whether the patient has arterial hypertension, obesity, or cancer. Once the data are submitted, the tool calculates the most probable outcome (moderate or severe COVID-19) and it provides the probability percentage of the prediction (Figure 5).

## 4. Discussion

Although several studies have been conducted with larger numbers of subjects using ML techniques [36,37,38], the patient sample in this study is unique in that it includes unvaccinated hospitalized and non-hospitalized patients. Vaccinated patients may be protected from the disease not because of their own traits but because of acquired immunity [39]. The data of the study were collected at the beginning of the pandemic when all patients were first infected with COVID-19 and therefore not immunized either by previous infection or by vaccination. Nevertheless, a study where vaccinated patients are intentionally excluded is still relevant today, as 2.2 billion people, mainly in developing countries, are unvaccinated [4] and vaccination is no longer considered mandatory in developed countries [40]. Furthermore, immunization either through vaccination or disease is not long-lasting [41]. Finally, vaccination protects only against established viral strains [42] and unvaccinated populations provide pools for the emergence of new variants. Therefore, the removal of immunized patients created an algorithm which shows the net effect of traits. Such a tool could be particularly useful in both developing and developed countries, as it distinguishes those individuals with a predisposition for severe COVID-19 outcomes, encouraging them to get vaccinated, as a preemptive measure. In addition, the inclusion of outpatients represents a large proportion of patients who have not been studied in the literature and may represent more than 40% of all infections [43]. As these patients were treated at home, they were not subjected to any clinical tests, therefore relevant parameters, such as ferritin, D-dimer, and glucose blood levels, were removed from the ML.

Our ML feature importance analysis highlighted five risk factors in descending order of significant prognostic value for the development of severe COVID-19: age, arterial hypertension, sex, obesity, and cancer. The statistical analysis verified these findings. A systematic review and meta-analysis of 17 studies with 44,672 COVID-19 patients found that age is associated with greater disease burden, longer ICU stay, and higher mortality [44]. Hypertension also increases the risk of ICU admission, mechanical ventilation, and death and has more adverse effects in those who do not take medication to control their blood pressure [44]. Angiotensin converting enzyme (ACE) inhibitors or angiotensin receptor blockers (ARBs), which were initially blamed for the poor outcome of the disease, do not affect the clinical outcome of patients with COVID-19 [45]. The observed preponderance of men in intensive care units was initially attributed to the effect of sex hormones on the immune system [46]. However, a recent meta-analysis refutes this explanation [47]. The influence of androgens on TMPRSS2 expression may provide a valuable scenario. In addition, as ACE2 is an X-chromosome gene and is strongly associated with the entry of SARS-CoV-2 into the human body, there may be a potential heterozygosity protection in women [48,49]. Other aggravating factors such as hypertension and poor health behavior have already been associated with men and need to be taken into account [13]. Hypotheses to explain the association between obesity and COVID-19 include mechanisms known to be induced by obesity, such as inflammation, immunosuppression, hypercapnia, and ACE2 overexpression [50]. A meta-analysis of 46 studies with 625,153 patients with COVID-19 confirmed that obese patients had a significantly higher risk of severe disease, intensive care unit admission, and death due to COVID-19 [51]. Cancer was also associated with greater disease burden, longer ICU stay, and higher mortality [52].

The nested cross-validation approach utilized in this study helps mitigate the bias–variance trade-off that is a key challenge in machine learning model development. By having separate inner and outer cross-validation loops, the inner loop tunes the hyperparameters to minimize the bias (underfitting) of the model on the validation sets. Meanwhile, the outer loop evaluates the generalization performance on the held-out test sets, providing an estimate of the variance (overfitting) of the model to unseen data. This nested approach reduces the risk of overfitting the hyperparameters to the test set, which could lead to an overly optimistic estimate of the model’s true performance. Additionally, techniques like feature selection via mRMR help reduce variance by removing redundant or irrelevant features that could overfit noise in the training data. Overall, this rigorous nested cross-validation framework, coupled with feature selection, aims to achieve a well-calibrated bias–variance balance for the final predictive model.

The statistical analysis identified additional risk factors, such as two comorbidities (diabetes mellitus and COPD) and two clinical tests (blood ferritin and glucose concentration). Diabetes mellitus is considered a risk factor for complications due to COVID-19 [53] and blood glucose levels are indicative of diabetes. COPD patients are at increased risk of COVID-19 severity [54]. Although diabetes mellitus and COPD are statistically significant comorbidities, their elimination from the ML-based prediction model though feature selection was due to the fact that they were correlated with other features in the model, and therefore, the model had already captured most of the information provided by them. Our study showed that blood ferritin levels on the first day of admission were almost three times higher on average in ICU patients than in those who were simply hospitalized. Other studies have also shown ferritin’s importance in non-intubated patients and in patients with thrombocytopenia in COVID-19 severity [55,56]. However, ferritin levels were not included in the ML analyses because there were no data for the patients of the “moderate” group who were not hospitalized.

The aim of the statistical analyses (χ^2^ and odds ratio) was to identify each risk factor of severe COVID-19. On the other hand, the aim of ML analysis (Linear Discriminant Analysis) was to identify those risk factors with the highest predictive power for the severity of the disease. In the feature selection process in ML analysis, some comorbidities of major importance for disease outcome, such as diabetes and COPD, were eliminated on the grounds that they could not improve the prediction performance of the algorithm, although the statistical analysis proved them significant.

A COVID-19 severity prediction algorithm combining simple patient demographic and comorbidity data could be an important prognostic tool contributing to the appropriate management of both patients and financial resources [57]. It is important to include more data to improve the predictive performance in supervised learning [58] and certainly to continue to include out-of-hospital and unvaccinated patients, although it is difficult to find these volunteers.

## 5. Conclusions

Following applied statistical and machine learning approaches, we identified risk factors associated with severe COVID-19, revealing that patient demographics (age and sex) and comorbidities (arterial hypertension, obesity, and cancer) had the highest predictive value using Linear Discriminant Analysis with mRMR and scaling. We developed a user-friendly web tool based on this model to predict COVID-19 severity using easy-to-acquire predictors. A characteristic of this tool is its design based on an unvaccinated population, minimizing biases and reflecting the virus’s pure impact on the human body. Data were collected between 12/2020 and 3/2022, allowing for estimations of prevalent virus strains during this period. In the future, the algorithm could be enhanced with additional secondary screening features such as gene profiles to improve prognosis. By having excluded complex information from specialized blood tests and extensive family history, an increase in the sample size with the inclusion of newly diseased patients could enable the tool to serve as an initial and quick predictor, empowering patients to gain insight into potential disease signs or warning high-risk individuals to get vaccinated. By including new patients, slight adaptations of the predictive model to the emerging viral variants may occur. Nevertheless, the current model may be resilient to the impact of any new variants [59], as the five selected features that the model relies on are very generic. Thus, it is unlikely that the current model may not apply to new variants.

## Figures and Tables

**Table 1:** Contingency tables of demographic variables (statistically significant findings in bold).

Variables	Moderate n (%)	ICU n (%)	*p*-Value	OR	99% CL
	204 (63.1%)	119 (36.8%)			
**Age (years)**	47.8 ± 15.8	66.6 ± 14.0			
<40	59 (28.9%)	4 (3.3%)	Reference		
>40	145 (71.1%)	115 (96.6%)	**<0.001**	11.70	2.97–45.97
**Sex**					
Female	108 (52.9)	34 (28.6%)	Reference		
Male	96 (47.1%)	85 (71.4%)	**<0.001**	2.81	1.49–5.30

**Table 2:** Contingency tables of comorbidities (statistically significant findings in bold).

Variables	Moderate n (%)	ICU n (%)	*p*-Value	OR	99% CL
	204 (63.1%)	119 (36.8%)			
**Obesity**					
NO	190 (93.1%)	96 (80.7%)	Reference		
YES	14 (6.9%)	23 (19.3%)	**0.0011**	3.25	1.28–8.24
**Diabetes mellitus**					
NO	185 (90.7%)	84 (70.6%)	Reference		
YES	19 (9.3%)	35 (29.4%)	**0.001**	4.06	1.80–9.10
**Dyslipidemia**					
NO	171 (83.8%)	85 (71.4%)	Reference		
YES	33 (16.2%)	34 (28.6%)	**0.0087**	2.07	1.01–4.24
**Thyroid Disease**					
NO	181 (88.7%)	95 (79.8%)	Reference		
YES	23 (11.3%)	24 (20.2%)	0.030	1.99	0.87–4.51
**Bronchial Asthma**					
NO	191 (93.6%)	110 (92.4%)	Reference		
YES	13 (6.4%)	9 (7.6%)	0.682	1.20	0.37–3.82
**COPD**					
NO	202 (99%)	106 (89.1%)	Reference		
YES	2 (1%)	13 (10.9%)	**0.001**	11.94	1.71–89.77
**Arterial Hypertension**					
NO	166 (81.4%)	48 (40.3%)	Reference		
YES	38 (18.6%)	71 (59.7%)	**<0.001**	6.46	3.13–12.59
**Atrial Fibrillation**					
NO	200 (98%)	107 (89.9%)	Reference		
YES	4 (2%)	12 (10.1%)	**0.003**	5.61	1.22–25.59
**Neurological Diseases**					
NO	193 (94.6%)	108 (90.8%)	Reference		
YES	11 (5.4%)	11 (9.2%)	0.190	1.79	0.57–5.59
**Cancer**					
NO	196 (96.1%)	102 (85.7%)	Reference		
YES	8 (3.9%)	17 (14.3%)	**0.001**	4.08	1.29–12.86
**Coronary Diseases**					
NO	198 (97.1%)	110 (92.5%)	Reference		
YES	6 (2.9%)	9 (7.5%)	0.066	2.70	0.67–10.85

**Table 3:** Contingency tables of blood tests of hospitalized patients on admission day (statistically significant findings in bold).

Admission Day	On a General Ward	ICU	*p*-Value	OR	99% CI
**Ferritin**	97	72			
Ferritin Mean	578.93 ± 561.65	1477.47 ± 1870.41			
Ferritin > 800	20 (20.6%)	36 (50%)	Reference		
Ferritin ≤ 800	77 (79.3%)	36 (50%)	**<0.001**	0.26	0.10–0.63
**D-dimers**	146	74			
D-dimers Mean	2.92 ± 12.57	2.67 ± 7.72			
D-dimers > 2.5	12 (8.3%)	15 (20.3%)	Reference		
D-dimers ≤ 2.5	134 (91.7%)	59 (79.7%)	0.012	0.35	0.12–1.03
**Glucose**	101	78			
Glucose Mean	126.21 ± 44.43	162.84 ± 70.33			
Glucose > 200	6 (5.9%)	16 (20.5%)	Reference		
Glucose ≤ 200	95 (94.1%)	62 (79.5%)	**0.005**	0.24	0.06–0.90

## Data Availability

Researchers wishing to access the data used in this study can make a request to the corresponding author.

## Supplementary Materials

The following supporting information can be downloaded at https://www.mdpi.com/article/10.3390/biotech13030022/s1, Table S1: Medians and means (in parenthesis) of performance metrics of various algorithm and hyperparameter combinations.
